# A comparison of traditional food and health strategies among Taiwanese and Chinese immigrants in Atlanta, Georgia, USA

**DOI:** 10.1186/1746-4269-9-61

**Published:** 2013-08-27

**Authors:** Sandy Jiang, Cassandra L Quave

**Affiliations:** 1 Center for the Study of Human Health, Emory University, 550 Asbury Circle, Candler Library 107, Atlanta, GA 30322, USA

**Keywords:** Traditional Chinese medicine, Medicinal foods, Migration, Traditional medicine, Intercultural healthcare

## Abstract

**Background:**

Ethnobotanical studies on the use of plants amongst migrant populations are of great relevance to public health. Traditional health strategies, which incorporate plants as medicines, foods, or both – can play an important role in individual well-being. However, at the same time, migrant populations’ traditional knowledge of such practices may be under a state of greater threat of decline due to factors such as limited access to the plant materials and physical isolation from the homeland, which serves as the primary living reservoir for this knowledge.

**Methods:**

In this study, we conducted a medical ethnobotanical survey focusing on a comparison of local medicinal food and health strategies with members of two Asian immigrant populations in metro-Atlanta: Chinese and Taiwanese. Snowball sampling techniques were employed to recruit 83 study participants, 57 of which were included in the final analysis. Semi-structured interview techniques were used to question participants about their beliefs and usage of the yin yang system, usage of Chinese herbs and medicinal foods, preference and usage of Eastern and Western medicines, and gardening for medicinal foods.

**Results and conclusion:**

Comparison of the two groups demonstrated a remarkable difference in health strategies concerning medicinal plant use, including statistically significant differences in beliefs concerning yin and yang, uses of Eastern versus Western medicine, and gardening for medicinal foods. Domestic health strategies in the form of medicinal foods play an important role in local health practices, especially among the Taiwanese participants. The collective desire for the use of both Eastern and Western medicine by both groups highlights the important role that cultural competency training will play in preparing allopathic health practitioners to serve increasingly diverse patient populations in the US.

## Background

Chinese and Taiwanese immigrants, like many other ethnic populations in the United States (US), heavily rely on herbal medicines and there is a growing interest in traditional practices of health care as a complement to biomedicine [1,2]. Traditional herbal medicines include “naturally occurring, plant derived substances with minimal or no industrial processing that have been used to treat illness within local or regional healing practices” [3]. Ethnobotanical studies concerning intercultural health practices amongst migrant populations can contribute to an emerging landscape of public health knowledge concerning immigrants in the US, and assist in informing health policy for the improvement of integration and access to biomedical services for these minority populations [4]. While many medical ethnobotanical studies have been dedicated to migrant health strategies in Europe (see e.g., [5-18]), relatively few have been dedicated to such studies of migrant populations in the US (see e.g., [19-26]). Moreover, although Traditional Chinese Medicine (TCM) is gaining acceptance as a major form of Complementary and Alternative Medicine (CAM) in the US, the use of medicinal foods by Chinese immigrants remains one of the least understood and neglected areas of anthropological research [27].

Traditional medical systems are incredibly important in the developing world, and in some parts of Asia, 80% of the population depends on it for their primary form of healthcare [28]. The World Health Organization (WHO) defines traditional medicine (TM) as “the sum total of knowledge, skills and practices based on the theories, beliefs and experiences indigenous to different cultures that are used to maintain health, as well as to prevent, diagnose, improve or treat physical and mental illnesses” and when adopted by other populations (outside its indigenous culture), TM is often termed CAM [28]. The heavy reliance on TM in the developing world is of critical importance to the economically developed nations that people migrate to as they bring with them a great diversity in health beliefs and strategies that are often quite different from the biomedical model. Allopathic practitioners are often lacking in the appropriate cultural competency training required for successful engagement with the great diversity of health modalities used by various migrant populations. However, there is one central facet common to most TM paradigms: the importance placed on the relationship between diet and health. Specifically, certain plants are purposely incorporated into the diet as a health strategy for the prevention and treatment of a wide range of medical conditions. Indeed, indigenous peoples have used foods in this manner for thousands of years for these purposes [29].

Since plants may be used in the role of medicine, food, or both – it is often difficult to draw a line of strict division between these two groups. Instead, the categorization of edible plants often falls somewhere along the line of a food-medicine continuum rather than into distinct, well defined groups. In previous work on wild health foods in the Mediterranean, Pieroni and Quave differentiated between plants used as medicines, foods, functional foods, and medicinal foods [30]. Specifically, whereas some plants are used in the strict context of either a food or a medicine, others may fall into the intermediate category of either a functional food or medicinal food. The main difference between the latter categories being that while functional foods are purposely incorporated into the diet for their general health benefit (i.e. cited as being “healthy”, “good for the blood”, or “depurative”), medicinal foods are eaten to fulfill a very specific medicinal role (i.e. for the treatment and/or management of chronic diseases like hypertension or diabetes). In the case of medicinal foods, oftentimes the plants must first be processed and detoxified prior to ingestion as the line between toxic and therapeutic dose may be thin. TCM uses this same concept with respect to yin and yang, and this is highlighted by the belief that too much of one becomes poisonous to the body. Thus, a major goal of TCM is to maintain a balance between the two opposing energy entities. TCM originated in ancient China more than 5,000 years ago and includes the use of herbs, acupuncture, and other methods, all aiming to promote the balance and harmony of the human body [31,32].

In the 1990’s, the majority of Chinese in the US were foreign born with strong cultural values, beliefs, and traditional health practices intact, which caused many to be apprehensive and unfamiliar with Western medicinal concepts, terminology, diagnostics, and treatments [27]. Despite the fact that the majority of Chinese immigrant health treatments were in the form of home folk remedies followed by a combination of Western and Chinese medicine, there existed a lack of culturally competent services for Chinese patients and thus this presented challenging problems to health care providers [27]. Although improvements have been made in the incorporation of CAM into the US medical system since the 1990’s, recognition and understanding of TCM practices as well as other domestic health practices still remains low among allopathic practitioners, and thus Chinese immigrants are often wary of disclosing cultural practices to their allopathic healthcare providers [27].

While the WHO has continued to support traditional medicine since the 1970’s, implementation of complementary medicine with allopathic medicine has proven challenging to most nations. Perhaps this is because of the strained institutional relationships between both traditional and allopathic healthcare systems [33]. Societal barriers to intercultural healthcare include low general acceptance by biomedical healthcare providers, which is compounded by issues regarding safety, efficacy, quality, and rational use of traditional medicine. Moreover, there is debate whether both medical systems should be integrated, or rather allowed to co-exist. Some contend that TM needs to be evaluated within its own framework rather than approved and subdued by rules of allopathic medicine [34]. This troublesome institutional relationship between TM and biomedicine stands in sharp contrast to its reality in the daily lives of many people, who often use both systems together and consider them as complementary rather than discrete systems [33,34]. Moreover, it is important to note that health matters are often viewed as a family coordinated activity rather than an individual responsibility [35,36]. For example, older Chinese patients will often rely on their social network as opposed to their health providers for health information, asking for home remedies and shared knowledge.

Allopathic medicine most strongly differs from TCM in that it focuses on Cartesian dualism, where body and mind are treated separately and most medical procedures are used for the treatment of physical diseases [37]. In contrast, concepts from TCM describe whole body homeostasis and the maintenance of it to remain well. While allopathic practitioners might seek to remove the immediate cause of illnesses, the traditional TCM practitioner tends to concentrate his efforts on helping to restore the body homeostasis using only body mechanisms [37]. Chinese food therapy teaches that different foods have varying quantities of yin and yang. Nutrition, for the Chinese, is based on the belief that food provides energy, also known as “chi”, but this does not mean energy in the limited Western sense [38], and thus, this medicinal teaching of yin and yang, or the balance of energies so as to avoid becoming poisonous is not recognized by Western scientific understanding [39]. Yin and yang in foods are described in terms of “heating” and “cooling”, which is a translation of the Chinese terms “shang huo” or “jiang huo”, literally meaning lowering or increasing heat. Thus, Chinese medicine focuses on balancing a correct diet in order to maintain this balance of heating and cooling chis. Maintaining a healthy balance of the two is essential to promoting good health. As such, the Chinese utilize particular foods and herbs in medicinal doses to alleviate imbalances in yin and yang.

While globalization and convenience have made processed foods more easily accessible, this has also resulted in some loss of traditional knowledge concerning food preparation (especially with regards to medicinal foods) among younger generations [40]. Today, many common ingredients to Chinese dishes can be found in supermarkets and ethnic markets in the US, but a considerable share, especially those of good quality, cannot be found or are available only at great expense. Limitations in access to ingredients can result in a decline in both their actual use and traditional knowledge of *how* to prepare and use them. Medicinal foods have experienced a decline in perceived value among younger generations as institutionalized medicine has become commonplace and food no longer serves in a major therapeutic role [40]. Rapid deterioration of this cache of knowledge makes it necessary to study and preserve local knowledge of medicinal foods.

The central question of our study was whether these two immigrant groups continue to use TCM (specifically in the context of medicinal foods) in similar ways, or whether sociocultural factors have led to shifting paradigms in health strategies concerning food and influenced levels of connectivity to the older ways of life practiced in their homeland. Most papers concerning immigrant health reflect the opinions of policy makers, physicians, healthcare facility managers, or traditional healers, instead of the opinions of a representative sample of community members [41]. Moreover, current literature often emphasizes collaborations with traditional healers, who hold specialist knowledge, whereas much less attention is paid to “domestic medicine” that takes place in households, particularly under the direction of non-specialist family members such as mothers and elderly family members [42]. Instead, this paper focuses on plants that are integral to traditional knowledge systems concerning household food and health strategies among Chinese and Taiwanese immigrants. Here, we aim to address differences in beliefs and usage of the yin yang system, usage of Chinese herbs and medicinal foods, preference and usage of Eastern and Western medicines, and gardening for medicinal foods between the two groups. Social, political, and cultural differences between the two groups are also discussed as they relate to diverging health practices within the Asian immigrant population in Atlanta.

### Study area

**Figure 1:**
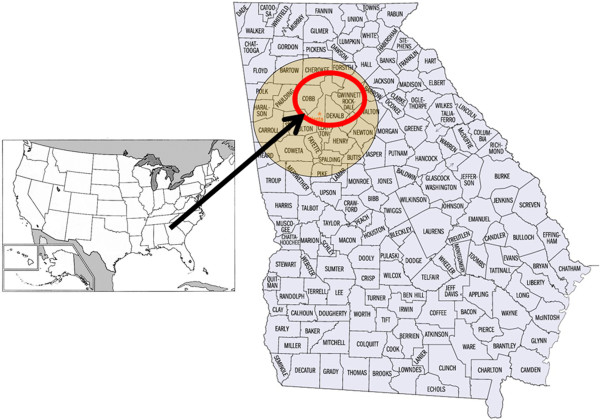
Map of the study area: Northeast Atlanta, Georgia, USA.

Metropolitan Atlanta is located between 33.7489° N and 84.3881° in the southeast of the United States in the state of Georgia (Figure 1). It has a humid subtropical climate with four seasons. Metropolitan Atlanta occupies a total area of 21,694 km^2^ and is one of the fastest growing regions in the United States, with a total population of ca. 5.5 million and an Asian population that comprises over 60% of Georgia’s total Asian population [43].

The Northeast Atlanta region consists of Cobb, Dekalb, Fulton, and Gwinnett counties, with Fulton and Gwinnett having the majority of the Chinese and Taiwanese populations (Table 1). While the increase in Chinese population after the mid 1960’s was a result of immigration laws, most Chinese leaders cite the main factors that really attracted Chinese immigration to Atlanta included an established Taiwanese government consulate and low cost of living and economic prospects [44]. Immigrants were at first concerned that they would have no access to their original foods found back in their home countries. However, they integrated to their new environment both in terms of culture and food with little difficulty as Atlanta was an international city and there were local Chinese grocery stores around Atlanta. Earlier immigrants had to incorporate or substitute American produce in their diets, but by the late 1980’s, a tight community of Chinese immigrants had sprung up in Chinatown in the Chamblee Dunwoody area including the Chinese Community Center, which is sponsored by the Taiwanese government [44]. It is interesting to note that the first wave of Chinese immigrants came for economic betterment, but the second generation in the 1980’s was comprised of students, scholars, and professionals who flocked to the US to pursue higher education or research. They were different from the earliest immigrants in that they tended to be more diverse and came from urban cities [27]. Immigration was difficult for Taiwanese until the 1965 Immigration Act, which increased the population of Asian immigrants, but it was not until 1980’s with the 1982 Immigration Reform Amendment was there an increase in Taiwanese immigrants. The reform set up a quota allowing 20,000 immigrants per year from Taiwan, separate from those of China [45]. Chinese schools and community centers have kept most Chinese traditions and cultures alive, but younger generations are still quickly assimilating to their surrounding American influences. Prices on once hard to obtain foodstuffs have decreased dramatically over the course of twenty years and today, with the large surge in Korean populations, Korean markets have competitively provided all foods, dry or fresh, in every imaginable form and low prices.

**Table 1:** Study population demographics

	**Georgia**	**Cobb County, GA**	**Dekalb County, GA**	**Fulton County, GA**	**Gwinnett County, GA**
**Total Asian**	320,905	31,164	37,095	52,213	86,957
**Chinese (except Taiwanese)**	44,604	4,961	5,767	10,025	10,624
**Taiwanese**	3,483	378	525	959	886

Although collectively, the Chinese are grouped under one population in US census records, they are divided politically, culturally, and socially into two groups, the Taiwanese and Chinese. Politically, the People’s Republic of China (PRC) is still under authoritarian control while Taiwanese citizens have lived under democracy since the 1980’s [46]. This dichotomy has led to political and social economic differences between the two groups. Even in the US, Chinese and Taiwanese cooking styles still remain distinct as well as usage of medicinal foods and TCM. Although much of the literature lumps these two groups together under one ethnic category as Chinese, most Taiwanese (including the participants in this study) argue that their culture differs much from their Chinese counterparts. Due to their cultural and political differences, we chose to analyze these two groups separately rather than combined into one ethnic group, with the hypothesis that their sociocultural differences may also influence food and health strategies as well.

## Methods

A total of 83 participants ranging in age from 18–68 years participated in this study. This included 22 students enrolled at Emory University, aged 18–23. Since students had limited opportunities to shop and prepare their own foods, choosing instead to eat at university cafeterias due to lack of travel access to local markets, they were excluded in the final data analysis. Among the 61 older participants, 30 identified themselves as Chinese and 27 as Taiwanese, and the remaining four as Singaporean or Malaysian. Thus, the 57 Taiwanese and Chinese were chosen for final analysis (average age of 44.7 for the Chinese and 52.5 years for the Taiwanese). Of the Taiwanese, 9 were female and 18 were male, while of the Chinese, 20 were female and 11 were male. All resided in the metro-Atlanta area of Georgia.

Institutional review board (IRB) approval was obtained prior to initiating the study (Emory IRB 00057325). Prior informed consent was obtained prior to all interviews and we adhered to the ethical standards of the International Society of Ethnobiology [47]. Participants were recruited using snowball sampling methods [48,49]. Semi-structured interviews were conducted using a set of 42 questions concerning medicinal food and health strategies were conducted in either English or Chinese, depending upon individual preference. Participants were questioned about their: 1) knowledge and use of medicinal plants (including parts, efficacies, preparations); 2) preference for Western or Eastern medicine; 3) belief and usage in yin and yang systems; and 4) gardening practices. Participants were also asked about the source of any plants used, the freshness and quality, as well as any substitutions for ingredients when the traditional Chinese materials were not available. Whenever possible, participants were asked to point out live specimens (especially for those that actively maintained gardens) and voucher specimens were collected for deposit in the Emory University Herbarium (GEO). In all other cases, pictures were used to verify the identity of commonly cited species. In addition to sampling from home gardens, trips were also made to Asian grocery stores and ethnic markets, where certain species were commonly acquired. Plant nomenclature follows the The Plant List database [50] and Angiosperm Phyologeny Group III system [51]. Chinese names cited by participants were verified using *Food Plants of China*[52] and the *Pharmacology of Chinese Herbs*[39].

### Study site

The location of interviews was at the convenience of the participant and varied from subject to subject. The majority of the interviews took place in the interviewee’s home, the interviewer’s home, or at local Chinese community centers. Some of the interviews were conducted in small groups with 2–3 participants while most of them were private one-on-one interviews. In both cases, all participants responded to the interview questions independently. The interviews all took place in NE Atlanta including Gwinnett, Fulton, and Cobb Counties. The cities included in the study included Duluth, Norcross, Roswell, Atlanta, Lawrenceville, Alpharetta, and Suwanee – all of which were chosen for their large Chinese immigrant populations. All participants had lived in this region for at least a year and were familiar with and regularly accessed local Chinese foods and produce.

### Data analysis

All data were input into a master excel sheet for statistical analysis. The Fisher’s approximation test was used to find statistically significant differences in the ethnobotanical knowledge and practices of two populations. Statistical significance was defined at a p-value of >0.05. Consensus indices are reported in Table 2 as a percent of total citations for each group.

**Table 2:** Plants used in the preparation of medicinal foods by Chinese and Taiwanese immigrants in the metro-Atlanta area

**Scientific name [Voucher ID]**	**Chinese name**	**English name**	**PU**^**a**^	**MC**^**b**^	**Medicinal use**	**%T**^**c**^	**%TW**	**%CHN**
AMARANTHACEAE								
*Amaranthus* spp. [SJ05]	苋菜	Amaranth	St; Le	SF; So	Source of iron	25	33	17
AMARYLLIDACEAE								
*Allium cepa* L. [SJ19]	洋葱	Onion	Bu	So; SF	High in nutrients; Antioxidant	19	30	10
*Allium chinense* G. Don [SJ04]	葱	Chinese scallion	Le	So; F; R	Immune enhancer	32	44	20
*Allium sativum* L. [SJ13]	大蒜	Garlic	Bu	SF; So	Immune enhancer; Used to treat first signs of cold/viral infection.	70	70	70
*Allium schoenoprasum* L. [SJ23]	韭菜	Chives	St; Le	Fr; Du	Tonic	18	19	17
APIACEAE								
*Angelica* spp. [SJH18]	当归	Angelica	Ro	T	Tonic	21	26	17
*Bupleurum chinense* DC.	柴胡	Thorowax root	Ro	T	Tonic	9	0	17
ARACEAE								
*Colocasia esculenta* (L.) Schott [SJ02]	芋头	Taro	Tu	So; Sm; St	Improve complexion; Remove toxins	28	41	17
ARALIACEAE								
*Panax ginseng* C.A. Mey. [SJH16]	人参	Ginseng	Ro	T; So	Maintenance; Warmth; Tonic	23	30	17
ASTERACEAE								
*Arctium lappa* L. [SJ17]	山瑶	Burdock	Ro	Fr; So	Immune maintenance & enhancer	28	37	20
AURICULARIACEAE								
*Auricularia auricula-judae* (Bull.) J.Schröt. [SJH03]	黑木耳	Black woodear	Fu	So	Improve circulation; Tonic	35	44	27
BRASSICACEAE								
*Isatis tinctoria* L.	板蓝根	Woad root	Ro	So	Maintenance; Immune enhancer	14	0	27
CARICACEAE								
*Carica papaya* L. [SJ03]	木瓜	Papaya	Fr	R	Digestive	14	30	0
CONVOLVULACEAE								
*Ipomoea aquatica* Forssk. [SJ10]	空心菜	Water spinach	St; L	SF; So	Tonic; Cooling	12	26	0
*Ipomoea batatas* (L.) Lam. [SJ09]	红薯	Sweet potato	Tu	So	Cold/flu remedy; Remove toxins	46	52	40
			L	So; Fr	Digestion; Remove toxins; Hypertension; Diabetes	16	33	0
CUCURBITACEAE								
*Cucurbita* spp.	南瓜	Pumpkin	Fr	So; D	Tonic; Remove toxins	18	22	13
*Momordica charantia* L. [SJ24]	苦瓜	Bitter melon	Fr	So	Cooling *chi* properties	32	44	20
*Momordica grosvenorii* Swingle [SJH07]	罗汉果	Monk fruit	Fr	So; T	Cooling *chi* properties; Anti-tussive; Expectorant; Sore throat remedy	32	37	27
FABACEAE								
*Astragalus onobrychis* L.	黄芪	Astragalus	Ro	T	Tonic	23	26	20
*Pueraria lobata* (Willd.) Ohwi [SJ20]	葛	Kudzu	L	Fr; So	Tonic	11	15	7
*Vigna radiata* (L.) R. Wilczek [SJH08]	绿豆	Mung bean	Fr	So	Cold/flu remedy; Remove toxins	53	63	43
GINKGOACEAE								
*Ginkgo biloba* L. [SJ18]	银杏	Ginkgo	Fr	So	Maintenance; Intelligence/heightened mental acuity	35	44	27
LILIACEAE								
*Lilium* spp.	百合	Lily	Bu	So	Maintenance; Immune enhancer	25	26	23
MALVACEAE								
*Abelmoschus esculentus* (L.) Moench [SJ08]	羊角豆	Okra	Fr	R; Sl; Dr	Diabetes (regulates blood sugar)	16	33	0
NELUMBONACEAE								
*Nelumbo nucifera* Gaertn. [SJ16]	莲	Lotus	Se; Ro	So	Immune enhancer; Tonic	37	44	30
PEDALIACEAE								
*Sesamum indicum* L. [SJH02]	黑芝麻	Black sesame	Se	R	Improve skin complexion; Tonic for the blood	46	52	40
POACEAE								
*Bambusa oldhamii* Munro [SJ24]	竹	Bamboo	St	F;So	Cooling *chi* properties	18	22	13
*Hordeum vulgare* L. [SJH01]	大麦	Barley	Se	So	Treat urinary tract infection; Fiber source; Improve digestion/laxative	30	37	23
RHAMNACEAE								
*Ziziphus jujuba* Mill [SJH10]	红枣	Red date	Fr	So	Improve circulation	53	63	43
ROSACEAE								
*Crataegus* spp.	楂	Hawthorn	Fr	R; Ca	Cough remedy; Cholesterol	32	37	27
*Pyrus bretschneideri* Rehder [SJ15]	鸭梨	Ya Li pear	Fr	So/Rs	Cough remedy	28	30	27
RUTACEAE								
*Citrus limon* (L.) Osbeck [SJ25]	柠檬	Lemon	Fr	J	Antibacterial; Antioxidant	12	15	10
*Citrus sinensis* (L.) Osbeck [SJH09]	橘子	Orange	Pe	T	Cough remedy	18	26	10
SOLANACEAE								
*Lycium chinense* Mill. [SJ01]	枸杞	Goji	Le; Be	So	Source of Vitamin A; Good for eyes and complexion	46	59	33
*Solanum lycopersicum* L. [SJ12]	西红柿	Tomato	Fr	R; So; SF; St	Immune enhancer; Male system	26	33	20
THEACEAE								
*Camellia sinensis* (L.) Kuntze [SJH05]	绿茶	Green tea	Le	T	Cooling *chi* properties	33	44	23
TREMELLACEAE								
*Tremella fuciformis* Berk. [SJH20]	英耳	White wood ear	Fu	So	Cooling *chi* properties	40	48	33
ZINGIBERACEAE								
*Zingiber officinale* Roscoe [SJ14]	姜	Ginger	Tu	So	Febrifuge	61	70	53

^a^Parts Used (PU): Be: berry; Bu: bulb; Fr: fruit; Fu: fungus; Le: leaves; Pe: skin peel; Ro: root; Se: seed; So/Rs: soup prepared with rock sugar; St: stems; Tu: tuber.

^b^Method of Cooking (MC): Ca: candied; D: dessert; Dr: drinks; Du: dumplings; F: fried; J: juice; R: raw; Sl: sliced; SF: stir fried; Sm: smashed; So: soup; St: steamed; T: tea.

^c^Total % (T): This reflects the total number of informant citations; TW%: percent of Taiwanese informants that cited this use; CHN%: percent of Chinese informants that cited this use.

## Results and discussion

### Belief and usage of Yin and Yang

Yin and Yang is the idea of balance of energies within a system, and in this study, subjects were first asked to say the first thing that came to their minds when they think of yin and yang. Afterwards, they were asked if they believed in the concept of yin and yang before being asked if they incorporated such beliefs into their food choices. Yin and yang in foods were described in terms of “heating” and “cooling”. The most popular connotations of yin and yang included man and woman, balance, and the ba kua symbol.

While there was a significant difference between Taiwanese and Chinese *belief* in yin and yang, there was no significant difference in the *usage* of yin and yang (Table 3). The Taiwanese were 14% more likely to believe in the energy system and were 13.3% more likely to have individuals who believed in some of the ideas as opposed to not believing at all. The Chinese were 19% more likely to reject the belief of yin and yang. While the raw data shows that the Taiwanese were 27% more likely to use yin and yang in their cooking, and Chinese were 23% more likely to not use the teaching, almost the same numbers between the two groups 11.1% for TW and 13.3% for CHN reported they encompassed some of the teachings. However, there was not enough evidence to conclude that Taiwanese were more likely to use the yin yang concept as compared to the Chinese. According to some Taiwanese participants, the fact that they were raised in an environment that highly promoted this belief of yin and yang made a lasting impression on them which kept this belief system in the back of their minds. However, they also stated that following migration to the US where the foods available to them are quite neutral in chi and are not as extreme in yin and yang as the original Asian foods, there was not a need to maintain this usage of yin and yang anymore.

**Table 3:** Responses to the survey questions concerning health beliefs and practices among Taiwanese and Chinese participants

	**Taiwanese**		**Chinese**		
	**n = 27**	**%**	**n = 30**	**%**	**p value**
**Belief in Ying and Yang**					0.010
Yes	19	70.4	17	56.7	
No	4	14.8	13	43.3	
Some	4	13.3	0	0.0	
**Usage of Ying and Yang**					0.093
Yes	19	70.4	13	43.3	
No	5	18.5	13	43.3	
Some	3	11.1	4	13.3	
**Medicinal use (Past)**					0.083
Western	8	29.6	18	60.0	
Eastern	10	37.0	6	20.0	
Both	9	33.3	6	20.0	
**Medicinal use (Present)**					0.013
Western	14	51.9	25	83.3	
Eastern	3	11.1	0	0.0	
Both	10	37.0	5	16.7	
**Gardening**					0.003
Yes	15	55.6	5	16.7	
No	12	44.4	25	83.3	

### Past and present usage of Western and Eastern medicine

Eastern medicine or “Zhong yi” was understood to mean TCM while Western medicine “xi yi” was understood to mean allopathic medicine. The participants were asked which system they preferred before being asked if they had used any other systems in the past when they were in their home countries. There was no significant difference in the past usage of Eastern or Western medicines. Individuals from both groups were almost equally likely to use Eastern medicine only or a combination of both Eastern and Western medicines. However, there was a significant difference (p = 0.013) in present preference for using Eastern, Western, or both medicines. All participants who cited that they would much prefer using only Eastern medicine over Western treatments were Taiwanese, while Taiwanese were more than twice likely to use a combination of both medicines as compared to the Chinese. When asked to comment on these findings, Taiwanese participants stated if they had the ability to choose – that is, if Eastern medicine was more established in Atlanta – they would have very much preferred it over Western therapies.

### Gardening, home gardens, fresh medicinal foods

While home gardens played a key role in giving people access to plants such as those described below, most medicinal food ingredients listed in Table 2 are normally purchased at local ethnic markets and international grocery stores found throughout the NE Atlanta area. In the context of this study, gardening entailed the planting of edible foods that served a medicinal purpose. It did not include flowers or ornamental vegetables, but rather vegetables that were grown specifically for health strategies. There was a significant difference between Taiwanese and Chinese immigrants’ experiences for gardening (p = 0.0027). The Taiwanese were three times more likely to plant gardens than the Chinese. Interestingly, there were some plants that Taiwanese planted that their Chinese counterparts did not. This included sweet potato leaves (*Ipomoea batatas* (L.) Lam), water spinach (*Ipomoea aquatica* Forssk.), and okra (*Abelmoschus esculentus* (L.) Moench) (Table 2).

In particular, sweet potato leaves are very popular among the Taiwanese and are praised for their ability to rid toxins in the body. Sweet potato leaves were cited as being very expensive at the store, and thus are a popular plant to have in food gardens where the sprouting sweet potato was usually buried in back yard soil and allowed to grow into vines that yielded dark heart shaped leaves on a vine.

**Figure 2:**
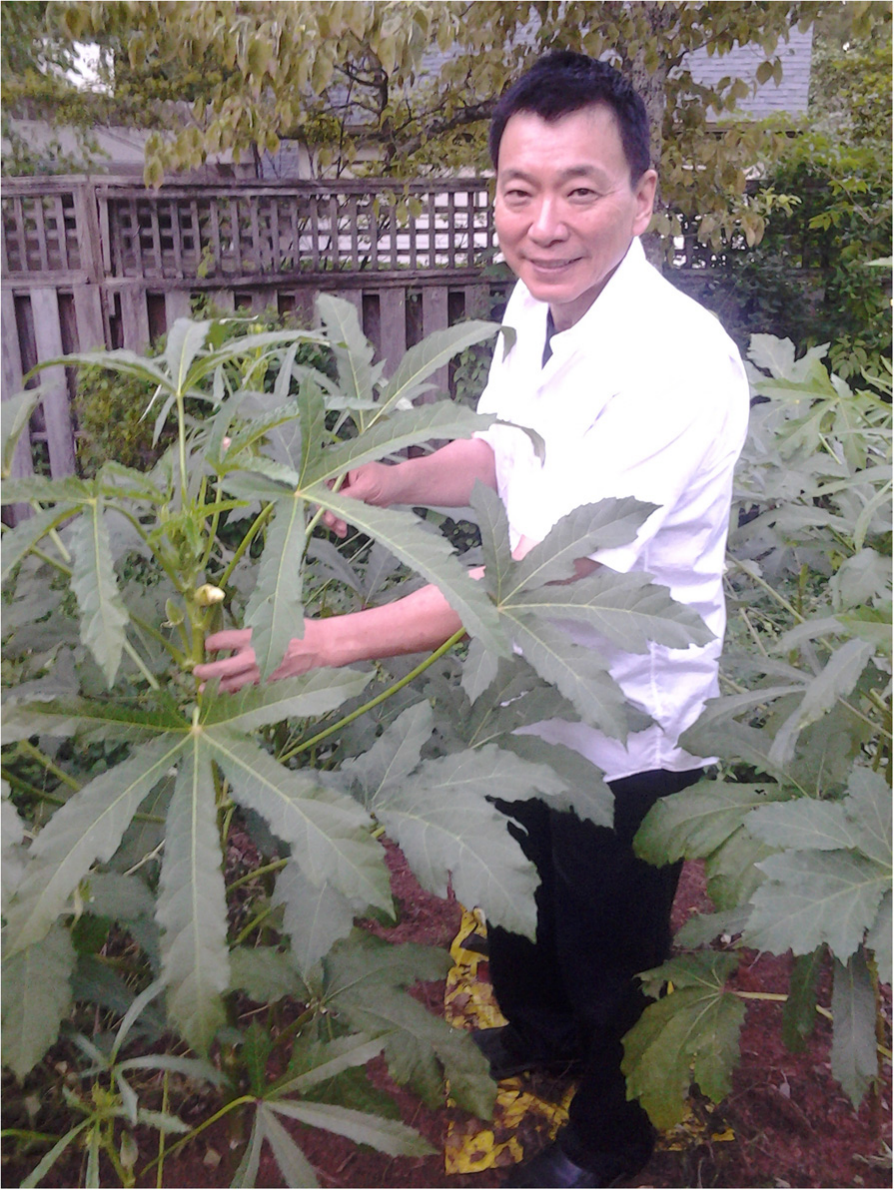
**Mr. Edward Sun with okra plants (*Abelmoschus esculentus *(L.) Moench) in his home garden.** Okra is a highly valued medicinal food commonly used in the management of blood sugar (anti-diabetes).

Another plant highly extolled by the Taiwanese gardening community was okra. Two of the study participants had at least seven plants in each of their gardens (Figure 2). One participant, who has diabetes, said that he relies on the okra to help level his blood sugar and pressure levels. “I cut the okra into eight pieces and place them in a cup of water for two days before I drink it down. It’s really good for my blood,” he states. Other participants (1/3 of the Taiwanese in the study) also agreed on the blood leveling effects of okra and recommend it to people suffering from blood sugar problems.

Water spinach, which is an invasive species especially if it comes into contact with bodies of water, is a highly prized plant consumed by many Chinese communities. While dishes containing this plant are usually found in restaurants, they are difficult to find in super markets due to their invasive status. One participant stated that he cannot figure out the mystery of how they arrive on restaurant plates: “Once, one of my neighbors tried to plant it and it spread and spread. He tried to wall up lotus roots too, but someone reported it, and no one has dared to try and plant it in water since then”. However, the plant can be found growing in soil in pots, which prevents their uncontrolled spreading.

### Frequency of medicinal food usage

**Figure 3:**
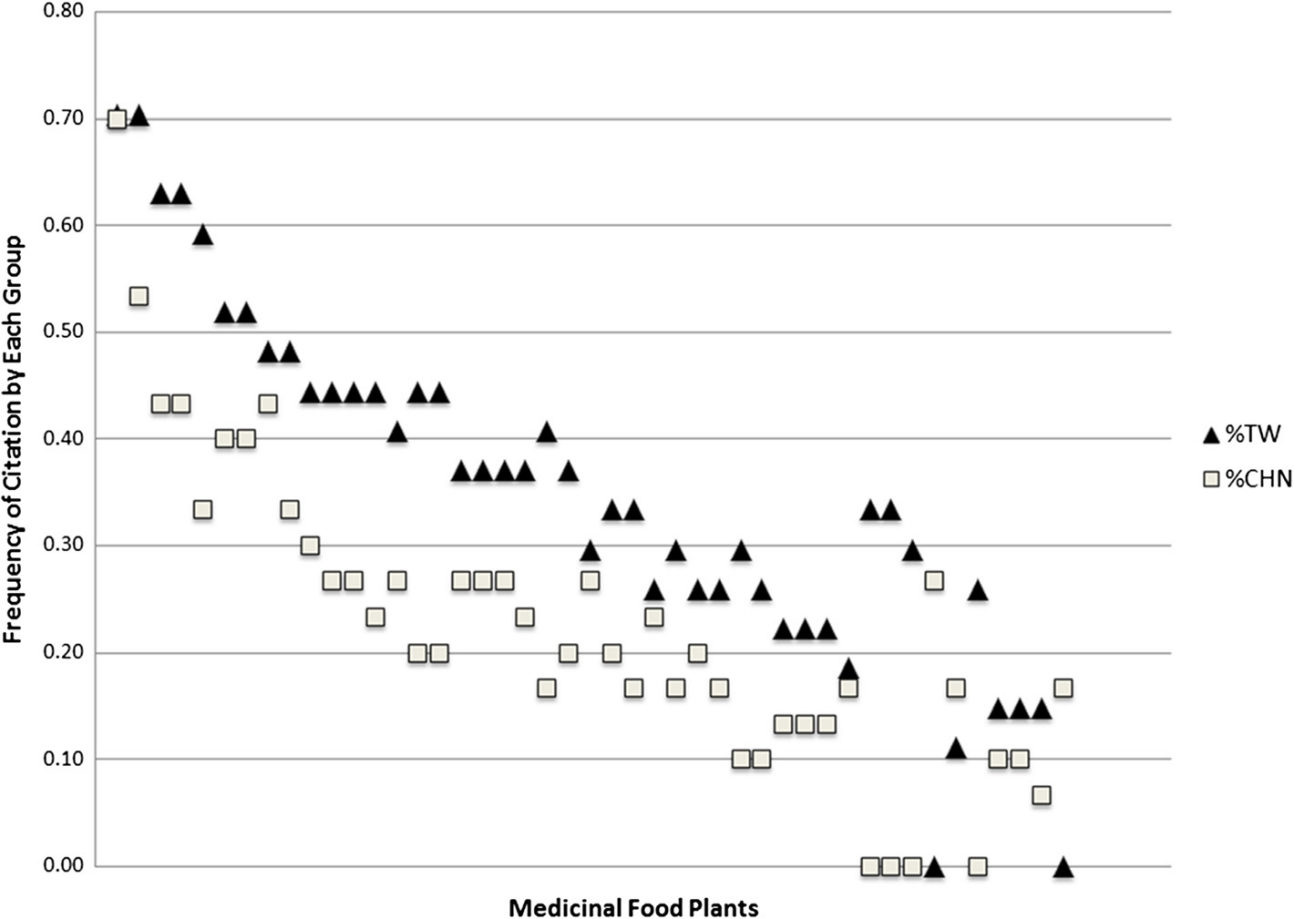
A comparison of frequency of citation of medicinal foods by Chinese and Taiwanese immigrants.

Table 2 shows the top 38 medicinal plants used, the part used, medicinal benefit, scientific name, Chinese and English names, and percentages of each population respectively that cited the plant, with the majority almost always having more Taiwanese usage (Figure 3). The medicinal uses reported in Table 2 were described within the paradigm of TCM, and are better clarified here. For example, some plants were used as “tonics, for ridding toxins, or immunity boosting”. “Detox remedies” were described in Chinese as “pai du” and are related to the idea of yin and yang for balancing the system’s chi, because an excess of chi is detrimental (or even considered poisonous) to the health. For example, when one has too much heating poison in the body, it is recommended that the person take a tonic or a medicinal plant that rid the body of toxins. Suitable medicinal foods for this purpose would include lotus (*Nelumbo nucifera* Gaertn) or mung bean (*Vigna radiata* (L.) R. Wilczek) soup, which would add cooling chi and return the body to a state of balance, or homeostasis. Note that there are two kinds of tonics, the heating and cooling kind, used for balancing the opposite poison. “Immunity boosting” foods are described as “bu”, which means that they provide nourishment or antioxidants for the body to use. For example, woad root (*Isatis tinctoria* L.) is used in this capacity. Foods described as being good for “maintenance” are similar to those described as having “tonic” properties, except that tonic medicinal foods are typically used only when there is a poison that must be rid of or a chi that must be counterbalanced. “Maintenance” is considered a milder form of a tonic in the sense that it is consumed daily but provides cleansing properties that are good for daily use to ensure the body is balanced and functioning properly. With regards to their placement of the food-medicine continuum [30], such foods used for daily health maintenance would fall under the category of functional, rather than medicinal foods.

Foods listed as having “heating chi properties or cooling chi properties” means that the foods themselves are mild to high in heating or cooling chis. The Chinese perceive heating chi foods to be “nutrient-dense”, and examples include chili peppers, red dates, and brown sugar. Nutrient-dense foods tend to include fried foods and those foods with an oily consistency, dense in minerals, or strong in color. These foods are usually high in iron and are consumed in the winter time or for those who are anemic and malnourished. Overconsumption of heating foods leads to nosebleeds, acne, sore throats, and constipation. Cooling foods are usually perceived to be less nutrient-dense and include green vegetables, and in particular for this study, mung beans, green tea (*Camellia sinensis* (L.) Kuntze) and white wood ear (*Tremella fuciformis* Berk.). These foods are normally consumed during the summer time, but are being increasingly used to counter balance Western foods which are described as having high heating properties for their oily, fried, and salty contents. Participants cited more cooling tonics are being used for their children than heating ones, explaining that there is a need for more cooling chi tonics to counterbalance the nutrient-dense diets that their children consume in the U.S. Overconsumption of cooling foods is believed to lead to opposite side effects of heating foods (e.g. diarrhea, coldness, and palor).

### Differences in Taiwanese and Chinese usage of medicinal food plants

The four plants unique to the Taiwanese participants included papaya (*Carica papaya* L.), sweet potato leaves (*Ipomoea batatas* (L.) Lam), water spinach (*Ipomoea aquatica* Forssk.), and okra (*Abelmoschus esculentus* (L.) Moench). All of these were planted in gardens, demonstrating the importance of these crops for them to be cultivated and well cared for by the families that use them. The Taiwanese prized the sweet potato leaves as a great tonic for digestion, the papaya for digestion, water spinach for tonic and cooling of the body, and okra for blood sugar maintenance.

The Chinese had two plants with medicinal food uses unique to their population: woad root (*Isatis tinctoria* L.) and thorowax root (*Bupleurum chinense* DC.). Both of these were proclaimed to be cure-alls (panacea) for all preventative illnesses. Chinese participants reported feeding their children a woad root soup to ward off colds and illnesses. When asked why they did not use a greater number of medicinal foods, most Chinese participants claimed that the effects were too slow and some expressed their doubts in the scientific reasoning behind the cooling/heating chi properties. It should be noted that three participants had professional (allopathic) medical experience. Two of the Chinese participants were fourth year medical students and one Taiwanese participant is a doctor practicing acupuncture in combination with Western medicine. All three discussed how they believed in the system when they were younger but their allopathic medical training had led them to prefer the Western practices.

### Use of other medicinal food products

In addition to the use of medicinal flora, other food products were cited as having important roles as medicinal agents. This included honey, brown sugar and vinegar. The applications and consensus indices for these remedies are reported in Table 4. Brown sugar was cited by both groups to be used for illnesses and symptoms associated with periods including alleviating menstrual cramps as well as providing nutrition. Participants explained that the brownness of the sugar was high in nutrition and helped nourish women from the blood loss during menstruation. Brown sugar was also described as being “warming” as it is high in nutrients compared to white sugar and thus falls in the category of “heating”. Honey was commonly cited as being used for the treatment of coughs and sore throats. Many participants also reported the use of a yali pear and honey drink to soothe irritated throats. White vinegar, or “bai chu” was described as being used for digestion and antibacterial purposes. It was often used in pickled vegetables.

**Table 4:** Other products used in the preparation of medicinal foods by Chinese and Taiwanese immigrants in the metro-Atlanta area

**Chinese name**	**English name**	**MC**	**Medicinal use**	**%T**^**a**^	**%TW**	**%CHN**
红糖	Brown sugar	Soup; Tea	Alleviation of menstrual cramps; Warming	14	11	17
蜂蜜	Honey	Soup prepared with ginger; Tea	Sore throats; Tonic	18	22	13
醋	White vinegar (acetic acid)	Condiment	Digestion; antibacterial	12	15	10

^a^Total % (T): This reflects the total number of informant citations; TW%: percent of Taiwanese informants that cited this use; CHN%: percent of Chinese informants that cited this use.

### Sociopolitical influences on immigrant health strategies

While all subjects were more or less comfortable with discussing their medicinal practices, it was noted that Taiwanese individuals talked significantly more (p-value = 0.045) and seemed more enthusiastic about sharing information about their health strategies (average interview time = 28.6 minutes). Chinese participants at times required more prompting, rephrasing of questions, or given more examples (average interview time = 22.4 minutes). Some seemed distracted and impatient about the process, and thus their interview times recorded were less than Taiwanese participants who had interviews that lasted up to 2 hours. Several participants provided clues that accounted for this difference in character. One reason may be that the interviewer is of Taiwanese descent, and there may have been less openness to one of different culture and background from the Chinese interviewees. Another reason why Chinese immigrants may be more hesitant on alternative medicine questions may be that they wanted to seem more literate in Western medicine. Most declined CAM usage or made hesitant remarks about usage of some medicinal foods. Some were quick to denounce Chinese medicine as if that is the right thing to do. One Chinese participant, upon hearing that we were interviewing Taiwanese people as well made a remark that the Taiwanese will definitely know more about this kind of stuff than us Chinese. “This kind of stuff” refers to medicinal plants and traditional Chinese medicine. One participant explained the reason why he received his acupuncture training and license in Taiwan rather than China was because of a political problem that the Chinese had which affected their views on TCM. Indeed, Taiwan is currently known as the TCM capital of the world. Heiner Fruehauf’s work, *Chinese Medicine in Crisis: Science, Politics, and the Making of TCM*, explains the suspicion that Chinese generally had towards TCM and those who practice it [53]. Fruehauf explains that when Western medicine entered China, TCM lost its position as being the one and only medicine “yixue” and became Chinese medicine “zhong yi”, defined in contrast to Western medicine “xiyi”. Attempting to fuse the two teachings has proven to be difficult. Chinese scholars were suspicious about political agendas, as the 1940–1970 era was unstable, and thus did not trust authority and lost confidence in medical authority as a consequence. Fruehauf points out a couple of main reasons why TCM has continued to have a slow comeback in Chinese culture. For one, none of the numerous TCM journals cover much traditional medical theory. Second, it is more profitable to order Western technologies, as reading pulses did not bring enough income into hospitals. Perhaps in a way, Chinese participants were wary about any socio-political agendas behind interview questions, and thus were hesitant in answering the questions. Fruehauf’s work echoes many of the sentiments expressed by the study participants. Future work will is necessary to further address this theory.

Yin and yang were regarded as important in both populations. Those who believed in yin and yang related the concept to balance and homeostasis. Significant disease results from disequilibrium of the positive yang principle and the negative yin principle and must be treated to restore the essential harmony between the pair. If the yin principle predominates owing to the deficiency of yang, then the former negative does not need to be inhibited but rather the positive needs to be built up [54]. In terms of food therapy, yin and yang was known as the heating and cooling system, which has been a very old and little modified philosophy. Dangerously heating foods, as agreed upon by general consensus, consists of foods that are high in nutrition and can only be consumed by the very healthy such as fried, long baked foods, and hot spices [38]. Mild heating foods, some of which were cited in this study, include red beans, ginger, ginseng and black wood ear, while cooling foods included many vegetables such as white wood ear, bitter melon, mung beans, and yali pears.

It is interesting to note that the participants who gardened the most were Taiwanese. They also maintained especially extensive gardens that spanned a whole back yard, for example, rather than a few potted plants. Taiwanese participants frequently called their nation the “King of Fruits and Vegetables” and proudly proclaimed their dedication in maintaining this tradition. While the Taiwanese participants interviewed represented almost all parts of Taiwan, the Chinese participants mostly came from urban settings, as those who left China in the late 1980’s were scholars or wealthier individuals seeking better economic opportunities. The majority of the Chinese participants came from Shanghai, Beijing, and Hong Kong and thus this urban background may have affected their responses, while the Taiwanese represented both urban and rural regions of Taiwan. One Taiwanese interviewee said, “Oh, yes, those Chinese immigrants are usually scholars. They know only books! Thus, they would know nothing about gardening and this whole Eastern medicine deal”.

## Conclusions

Ethnobotanical studies are incredibly important to public health as many migrant populations still rely on strategies which incorporate traditional knowledge of herbal remedies and medicinal foods for management of their health. It is projected that ethnic minorities will comprise the majority of the US population by 2050 [55], signifying a demographic trend that represents an increase in cultural diversity [56]. Addressing the health needs of this increasingly diverse population has become a progressively more visible public policy goal [57]. Migrants bring their own cultural beliefs about symptoms and underlying causes of illnesses, and preferred cultural treatments for these illnesses [58], including the practice of using medicinal plants imported from their home countries [19,21] and the use of local plants as substitutes for the food or therapy when the original ingredients are inaccessible or difficult to obtain [59].

This study presents a comparative analysis of two seemingly similar but socially and politically disparate ethnic immigrant groups and their usage of medicinal foods, traditional medicine preferences, and gardening habits. The results illustrate the medical system preferences, medicinal food usage, beliefs of yin and yang, and gardening habits among Taiwanese and Chinese immigrant populations in Atlanta. We have documented that among these immigrant populations, there is a great interest in incorporating traditional practices of health care (encompassing both folk medical practices and TCM) into the larger biomedical system. This was made most evident by the large number of those in the Taiwanese community who preferred to use both Eastern and Western medicine. Therefore, it is very important that physicians, particularly those who have many Chinese and/or Taiwanese immigrant patients, to understand the folk uses of these plants and be aware of possible synergistic effects or other herb-drug interactions that may occur when consumed in large quantities and in combination with Western pharmaceuticals [60]. As allopathic and CAM modalities each have their own unique merits, CAM requires more attention in the US biomedical oriented health care system. Ideally, allopathic physicians should be able to better understand and respond appropriately to questions raised by immigrant patients. This is especially relevant today in the context of the current shift towards a preference for a mixture of Eastern and Western medicine compared to the past where the majority of treatments were home remedies based off of TCM theory concerning yin and yang [27]. How an allopathic healthcare provider reacts to traditional beliefs from a patient will greatly influence the quality of the clinical encounter. For migrant populations in particular, it is crucial that the biomedical system recognize cultural values as the key to delivering culturally-sensitive healthcare. This includes the necessity of allopathic physicians becoming aware of commonly held folk medical beliefs in a community, as well as employing non-judgmental inquiries into their patients’ choice of adhering to ethnomedical beliefs and behaviors [61]. An increase in cultural competence training for biomedical practitioners (e.g. medical students, residents, nurses and other healthcare providers) could be useful in establishing a more trusted physician-patient relationship with migrant populations [62,63].

From a cultural standpoint, TCM serves as a resource for both the Taiwanese and Chinese communities to claim their cultural identity. Since TCM has always been a part of their lives, continuing the practice of TCM allows these older generations to perform and affirm their identity. TCM, most importantly to elderly parents, serves as a way to educate their children about the health knowledge and values that are deeply intertwined with the Chinese way of life [64]. It is perceived as a valuable resource for immigrant children to connect with their cultural roots as well as keep their culture alive across generations in a foreign land [64].

With China rising in economic power, much of the Chinese population resides in Westernized urban centers which may be linked to a decline in the usage of Chinese medicine and home remedies (including medicinal foods) which integrate concepts of yin and yang. TCM may be well integrated in the Chinese health system, but it still faces many challenges including a lack of tangible scientific results, loss of traditional knowledge across generations due to historical evolution and cultural changes, popularity of Westernization and modernization among Chinese people due to recent economic reform, and contamination with tainted products [65]. In the present study, many of the Chinese participants were from major cities and cited their disbelief in what many termed as an “archaic” and “slow” medicinal system and voiced their preference for the “faster” and “stronger” Western medicine. Many of them either gave up their traditional Chinese medicinal beliefs, or did not pass on their knowledge to their children, as shown in interviews among students and participants under the age of 23. While the Taiwanese were more likely to hold onto their traditional Chinese medicinal beliefs, they also did not pass down all of their medicinal knowledge to their children. Overall, both Taiwanese and Chinese immigrants stated that while they used TCM early in their children’s lives, they stopped when the children began to express preference for allopathic medicine. This disconnect with TCM amongst the younger generations was likewise supported by the interviews with the younger study participants, who reported a very limited understanding and appreciation for traditional Chinese medicines and medicinal foods.

It was interesting that in this study, only a few traditional medicinal herbs were regularly used (e.g. woad root, thorowax, astralagus, and angelica). This is in stark contrast to the fact that TCM practitioners commonly make use of hundreds of additional species that were not mentioned by either the Chinese or Taiwanese participants in the study. This may be an indication of integration into North American culture; more individuals are more willing to give up their traditional practices even though the herbs are increasingly becoming available with the boom in international and ethnic super markets. An alternative explanation is that this knowledge of herbal medicines is more restricted to TCM specialists, whereas medicinal foods play a more integral role in household folk remedies. The importance of medicinal foods in domestic health strategies should not be underplayed. Indeed, many self-limiting illnesses such as the common cold, flu, respiratory infections and uncomplicated diarrhea can often be successfully managed at the household level through the use of home remedies [66]. Likewise, other long-term, chronic illnesses such as diabetes and arthritis are also commonly managed with herbal home remedies in many parts of the world with most rural areas placing large emphasis on reproductive health and nutrition within household care [33,67]. In conclusion, domestic medicine, which may entail the use of herbal medicines, functional foods, or medicinal foods [30], should be a priority area for future research and health policy and intervention programs, especially for those concerning migrant communities in the US.

## Abbreviations

CAM: Complementary and alternative medicine; IRB: Institutional review board; TCM: Traditional Chinese medicine; TM: Traditional medicine; US: United States; WHO: World health organization.

## Competing interests

The authors declared that they have no competing interests.

## Authors’ contributions

SJ performed all interviews, conducted statistical analysis of the data, and drafted the paper. SJ and CQ conceived of and designed the study. CQ contributed to the overall scientific discussion. Both authors read and approved the final manuscript.
